# Characteristics of a global classification system for perinatal deaths: a Delphi consensus study

**DOI:** 10.1186/s12884-016-0993-x

**Published:** 2016-08-15

**Authors:** Aleena M. Wojcieszek, Hanna E. Reinebrant, Susannah Hopkins Leisher, Emma Allanson, Michael Coory, Jan Jaap Erwich, J. Frederik Frøen, Jason Gardosi, Sanne Gordijn, Metin Gulmezoglu, Alexander E. P. Heazell, Fleurisca J. Korteweg, Elizabeth McClure, Robert Pattinson, Robert M. Silver, Gordon Smith, Zheyi Teoh, Özge Tunçalp, Vicki Flenady

**Affiliations:** 1 Mater Research Institute – The University of Queensland (MRI-UQ), Brisbane, Australia; 2 International Stillbirth Alliance, Bristol, UK; 3 School of Women and Infants Health, Faculty of Medicine, Dentistry and Health Sciences, University of Western Australia, Perth, Australia; 4 Department of Reproductive Health and Research, World Health Organization, Geneva, Switzerland; 5 Murdoch Childrens Research Institute, Melbourne, Australia; 6 The University of Groningen, University Medical Center Groningen, Groningen, The Netherlands; 7 Department of International Public Health, Norwegian Institute of Public Health, Oslo, Norway; 8 Perinatal Institute, Birmingham, UK; 9 Institute of Human Development, Faculty of Medical and Human Sciences, University of Manchester, Manchester, UK & St. Mary’s Hospital, Central Manchester University Hospitals NHS Foundation Trust, Manchester Academic Health Science Centre, Manchester, UK; 10 Department of Obstetrics and Gynaecology, Martini Hospital, Groningen, The Netherlands; 11 Research Triangle Institute, Research Triangle Park, NC USA; 12 Department of Obstetrics and Gynaecology, University of Pretoria, Pretoria, South Africa; 13 University of Utah Health Sciences Center, Salt Lake City, USA; 14 National Institute for Health Research, Biomedical Research Centre & Department of Obstetrics & Gynaecology, Cambridge University, Cambridge, UK

**Keywords:** Stillbirth, Perinatal death, Neonatal death, Classification, Systems, Causes of death

## Abstract

**Background:**

Despite the global burden of perinatal deaths, there is currently no single, globally-acceptable classification system for perinatal deaths. Instead, multiple, disparate systems are in use world-wide. This inconsistency hinders accurate estimates of causes of death and impedes effective prevention strategies. The World Health Organisation (WHO) is developing a globally-acceptable classification approach for perinatal deaths. To inform this work, we sought to establish a consensus on the important characteristics of such a system.

**Methods:**

A group of international experts in the classification of perinatal deaths were identified and invited to join an expert panel to develop a list of important characteristics of a quality global classification system for perinatal death. A Delphi consensus methodology was used to reach agreement. Three rounds of consultation were undertaken using a purpose built on-line survey. Round one sought suggested characteristics for subsequent scoring and selection in rounds two and three.

**Results:**

The panel of experts agreed on a total of 17 important characteristics for a globally-acceptable perinatal death classification system. Of these, 10 relate to the structural design of the system and 7 relate to the functional aspects and use of the system.

**Conclusion:**

This study serves as formative work towards the development of a globally-acceptable approach for the classification of the causes of perinatal deaths. The list of functional and structural characteristics identified should be taken into consideration when designing and developing such a system.

## Background

An estimated 5.5 million perinatal deaths occur worldwide each year, many of which are preventable [1]. These deaths have enduring psychosocial consequences for parents, families and clinicians, with wide-reaching impacts on communities and society as a whole [2]. Perinatal deaths include both stillbirths (defined for international comparisons as fetal death from 28 weeks’ gestation onwards) and neonatal deaths (defined as death within the first 28 days of life) [3]. The recently proposed United Nations Sustainable Development Goals (SDGs) include a goal to end preventable deaths of newborns and children under 5 years of age by 2030 [4]. As neonatal deaths make up 44 % of the global under-five mortality rate [5], substantial progress towards this goal will not occur without reductions in neonatal deaths. Stillbirths, too, require accelerated attention. An estimated 2.6 million babies will be stillborn in 2015 [3], with further deaths in earlier pregnancy.

At the heart of prevention of perinatal deaths is understanding of their causes within a given population. Perinatal death classification systems aim to meet this need by allowing analysis of the factors leading to stillbirth within a population, but the use of multiple, incongruent systems hampers comparison of the underlying causes of perinatal death in different regions of the world [6–9]. A recent systematic review of perinatal death classification systems reported that 81 new or modified classification systems were used between 2009–2014 across 40 countries [8]. These systems differ in many important ways, including the number of primary causes recorded; whether an underlying cause is recorded; whether both stillbirths and neonatal deaths are included; whether antepartum stillbirths are distinguished from intrapartum stillbirths; and whether International Classification of Disease (ICD) codes are used [8].

The ICD is currently the only global system for classifying perinatal deaths [10]. There are however some limitations in the existing scope of the perinatal cause of death codes. In addition to this, the maternal condition contributing to the perinatal death is coded differently to when the same maternal condition results in a maternal death [11], which may make the system less desirable to users. While the current ICD (ICD-10) [11] includes extensive coding for newborns, it does not consistently treat the fetus as a distinct entity with its own diseases to be recorded separately from the mother [10]. Because of the potential loss of such information, ICD-10 has only limited utility for the accurate classification of stillbirths.

A single, uniform approach to the classification of perinatal deaths is critical to inform policy formulation and program implementation for the prevention of these deaths. While the need for such a system is not in dispute, there is no global agreement on the necessary characteristics of a universal perinatal death classification system. Desirable characteristics of perinatal death classification systems have been reported [10, 12], but these have not involved wide-reaching expert consultation and agreement. The World Health Organisation (WHO), and collaborating partners, is developing the WHO Application of ICD-10 to perinatal deaths: ICD-Perinatal Mortality (ICD-PM) [13]. The aim of this study was to inform the WHO’s work by establishing expert consensus on the essential characteristics of a high-quality global classification system for perinatal deaths. Our study therefore serves as formative research for the development of a globally-acceptable classification approach for perinatal deaths, from a systems user perspective.

## Methods

### Study design

In this study a Delphi consensus methodology was adopted. The Delphi technique is useful for research questions where the aim is to reach consensus from a field of experts, when there is no definitive “right” answer [14]. As part of this technique, we undertook three consultation rounds with responses in each round aggregated and fed back to the group [15].

### Assembling of expert panel

One hundred and eighty panel members were selected based on their expertise in perinatal death classification. We aimed to include panel members to ensure global coverage, as well multi-disciplinary expertise across relevant disciplines. The invited members were either authors of published peer-reviewed international journal articles in the field as identified in a comprehensive systematic review of the literature between 2009 to 2014[8], or recommended for invitation by other panel members. Panel members were initially contacted via email introducing the study and inviting them to participate. All panel members were invited to participate in subsequent consultation rounds, regardless of non-participation in Round 1.

### Data collection

Data were collected via a series of purpose-built online surveys using the software Checkbox® 6. We adopted a ‘quasi-anonymous’ Delphi technique [16], where each panel member was aware of the members forming the panel, but individual responses were kept anonymous.

### Procedures

#### Consultation round 1

In the first consultation round, a single open-ended question was posed, where the participants were asked to list all characteristics they considered important for a global classification system for perinatal deaths (Appendix 1). There were no word limit restrictions on response. Responses were collated and summarised in Microsoft Excel. Based on the open-ended responses, we developed a list of statements describing the optimal attributes of a global classification system – hereafter “*system characteristics*” (e.g. *“A global system must have a small number of main categories of causes of death”*, *“A global system’s causes of death must be mutually exclusive (not overlapping)”.* All characteristics and comments provided by the panel members were addressed. We combined characteristics that were identical or that varied only slightly. Comments and explanations provided by panel members associated with each system characteristic were included as “panel comments”.

#### Consultation round 2

In the second consultation round we presented the proposed system characteristics to panel members for evaluation. Panel members were asked to indicate their level of agreement with each statement via a five-point Likert scale (1 = Strongly disagree; 2 = Disagree; 3 = Neither agree nor disagree; 4 = Agree; 5 = Strongly agree). A textbox for more detailed qualitative feedback was also included for each characteristic. All responses were summarised in Microsoft Excel. For each system characteristic, we calculated the combined percentage of “agree” and “strongly agree” responses. Based on participant feedback, some characteristics were combined if they were very similar, and some were slightly re-worded.

#### Consultation round 3

In the third consultation round the system characteristics from the second round were organised into three groups:

- group 1: system characteristics agreed or strongly agreed with by ≥80 % of panel members;
- group 2: system characteristics agreed or strongly agreed with by 70–79 % of panel members; and
- group 3: system characteristics agreed or strongly agreed with by <70 % of panel members.

Panel members were asked to provide their opinion to either retain or discard each of the system characteristics in groups 1 and 2. We divided these system characteristics into “structural” characteristics and “functional” characteristics to facilitate scoring for panel members. Each characteristic was presented with level of agreement from Round 2. A textbox for further comments was provided for each characteristic. For system characteristics in group 3, there was no opportunity to discard or retain each characteristic, but one combined textbox was included to enable feedback for this group. Responses were summarised in Microsoft Excel and the percentage of participants that agreed to retain each characteristic was calculated.

## Results

### Characteristics of expert panel

A total of 180 experts were invited to participate in each consultation round. The number of panel members participating in rounds 1, 2 and 3 were 71, 52 and 51 respectively (39, 29 and 28 %). There were 80 unique panel members responding over the course of the study, with responding panel members generally overlapping across the consultation rounds. Twenty-one countries were represented by the panel members in Round 3 (see Table 1). Nine primary areas of expertise were represented, and four additional areas were represented as “other area” (Table 1).

**Table 1:** Country of residence and areas of work (“primary” and “other”) as identified by panel members in Round 3

Panel characteristics	Total *N* = 51
	Frequency (%)
Country of residence	
Australia	3 (5.9)
Bangladesh	2 (3.9)
Canada	1 (2)
China	1 (2)
Croatia	2 (3.9)
Ethiopia	1 (2)
India	4 (7.8)
Israel	1 (2)
Italy	2 (3.9)
Nepal	3 (5.9)
Netherlands	2 (3.9)
New Zealand	1 (2)
Norway	3 (5.9)
Pakistan	2 (3.9)
South Africa	4 (7.8)
Sudan	1 (2)
Switzerland	2 (3.9)
Turkey	2 (3.9)
United Kingdom	5 (9.8)
United Republic of Tanzania	1 (2)
United States of America	8 (15.7)
Primary area of work	
Database	1 (2)
Epidemiology	8 (15.7)
Neonatal nursing	1 (2)
Neonatology	6 (11.8)
Obstetrics	15 (29.4)
Paediatrics	4 (7.8)
Pathology	2 (3.9)
Policy/Programs	2 (3.9)
Public Health	12 (23.5)
Other area of work^a^	
Epidemiology	9 (17.6)^b^
Gynaecology	1 (2)
Midwifery	1 (2)
Neonatology	3 (5.9)
Obstetrics	7 (13.7)
Paediatrics	2 (3.9)
Pathology	2 (3.9)^c^
Perinatology	1 (2)
Policy/Programs	3 (5.9)^d^
Public Health	12 (23.5)^e^
Research	1 (2)^f^

^a^Other area of work given by 42 participants. Percentages calculated based on total sample of 51 participants

^b^Specifically: pregnancy outcomes; perinatal epidemiology; perinatal health surveillance; NCD; maternal and newborn health; teaching and research; perinatal surveillance; obstetrics; neonatal infections in developing countries; research data management; maternal and neonatal mortality.

^c^Specifically: perinatal pathology.

^d^Specifically: maternal and child health; maternal and perinatal health; emergency obstetric care; maternal and perinatal audits; health policy.

^e^Specifically: urban health; mortality review; community medicine; newborn health; medical birth and perinatal death register; infectious diseases; policy decision making; health systems; health informatics; reproductive health; maternal and child health; clinical patient care; teaching; perinatal; reproductive; maternal and perinatal health care; community-based newborn care; infant and pre-school child health care; global health; health systems; neonatology; immunity programs; stillbirth.

^f^Specifically: intra-uterine fetal death.

Percentages may not equal 100 due to rounding

### Consultation Round 1: Open-ended question

Responses to the initial open-ended question were collated into 46 system characteristics (see Table 2, column “Round 1 – Proposed characteristics”). The proposed system characteristics addressed a range of system properties, including whether the system should be hierarchical; whether associated conditions should be included; whether both stillbirths and neonatal deaths should be incorporated; and whether the system should allow more than one cause of death to be recorded. Other responses reflected practical requirements of a global system, including whether it can link to birth registries; whether it should be available in multiple languages; and whether it should link to relevant birth registries (see Table 2).

**Table 2:** Refinement of characteristics and inclusion agreement (% of panel members in agreement) with proposed characteristics in each round of the Delphi

Round 1 (*N* = 71) proposed characteristics	Round 2 (*N* = 52) agreement with system characteristic (%)	Round 2 proposed characteristics and notes on changes	Round 3 (*N* = 51) agreement to retain (%)	Round 3 preliminary proposed characteristics
1. A global system must have clear guidelines for use	98	A global system must have clear guidelines for use and definitions for all terms used	100	(F1) A global system must have clear guidelines for use and definitions for all terms used
2. A global system must produce data that can be used to inform strategies to prevent perinatal deaths	96.1	A global system must produce data that can be used to inform strategies to prevent perinatal deaths	96	(F2) A global system must produce data that can be used to inform strategies to prevent perinatal deaths
3. A global system must provide clear definitions for all terms used	96.1	Incorporated into #1	-	-
4. A global system must produce data that are easily understood and valued by end-users (those that use the cause of death data)	96.1	Incorporated into #8	-	-
5. A global system must be available in multiple languages	96.1	Incorporated into #10	-	-
6. A global system must be able to work with all levels of data (from both low-income and high-income countries)	94.1	A global system must be able to work with all levels of data (from both low-income and high-income countries), including minimal levels	98	(S1) A global system must be able to work with all levels of data (from both low-income and high-income countries), including minimal levels
7. A global system must allow easy access to the data by the end-users	94.1	A global system must allow easy access to the data by the end-users	92	(F3) A global system must allow easy access to the data by the end-users
8. A global system must be easy to use by those classifying the causes of death	92.6	A global system must be easy to use, and produce data that are easily understood and valued by users	100	(F4) A global system must be easy to use, and produce data that are easily understood and valued by users
9. A global system must have high inter- and intra-rater reliability	92.2	A global system must have high inter- and intra-rater reliability	94	(F5) A global system must have high inter- and intra-rater reliability
10. A global system must be available in different formats including inexpensive ehealth and mhealth options	92.2	A global system must be available in different formats including inexpensive ehealth and mhealth options, and in multiple languages	94	(F6) A global system must be available in different formats including inexpensive ehealth and mhealth options, and in multiple languages
11. A global system must distinguish clearly between causes of death and associated factors	90.6	Incorporated into #19	-	-
12. A global system must require neonatal deaths to be clearly distinguished from stillbirths	88.7	A global system must require neonatal deaths to be clearly distinguished from stillbirth	94	(F7) A global system must require neonatal deaths to be clearly distinguished from stillbirths
13. A global system must distinguish between antepartum and intrapartum conditions	88.7	A global system must distinguish between antepartum and intrapartum conditions	90	(S2) A global system must distinguish between antepartum and intrapartum conditions
14. A global system must be useable with minimal data	88.2	Incorporated into #6	-	-
15. A global system must include cause of death categories that are relevant in all settings	88.2	Incorporated into #16	-	-
16. A global system must use valid causes of death categories	84.9	A global system must ensure cause of death categories are relevant in all settings	96	(S3) A global system must ensure cause of death categories are relevant in all settings
17. A global system must have rules to ensure valid assignment of the cause of death	83	A global system must use rules to ensure valid assignment of causes of death	98	(S4) A global system must use rules to ensure valid assignment of cause of death categories
18. A global system must identify the underlying cause of death	83	A global system must identify the underlying cause of death	78	-
19. A global system must require associated factors to be recorded	81.1	A global system must require associated factors to be recorded and clearly distinguished from causes of death	94	(S5) A global system must require associated factors to be recorded and clearly distinguished from causes of death
20. A global system must allow more than one cause of death to be recorded	80.8	A global system must allow more than one cause of death to be recorded	78	-
21. A global system must require the single most important factor leading to the death to be recorded	78.9	A global system must require the single most important factor leading to the death to be recorded	86	(F8) A global system must require the single most important factor leading to the death to be recorded
22. A global system must have multiple levels of causes of death	77.4	Incorporated into #25	-	-
23. A global system must require both primary and secondary causes of death to be recorded	76.9	A global system must require both primary and secondary causes of death to be recorded	73	-
24. A global system must link to relevant birth registries	74.5	A global system must link to relevant birth registries	55	-
25. A global system must have a small number of main categories of causes of death	74.1	A global system must have multiple levels of causes of death, with a small number of main categories	82	(S6) A global system must have multiple levels of causes of death, with a small number of main categories
26. A global system should record the level of data available to assign the cause of death (e.g. verbal autopsy only, placental histology, autopsy, etc.)	73.6	A global system should record the level of data available to assign the cause of death (eg verbal autopsy only, placental histology, autopsy, etc.)	96	(S7) A global system should record the level of data available to assign the cause of death (e.g. verbal autopsy only, placental histology, autopsy, etc.)
27. A global system must incorporate both stillbirths and neonatal deaths	73.6	A global system must incorporate both stillbirths and neonatal deaths	86	(S8) A global system must incorporate both stillbirths and neonatal deaths
28. A global system must include a sufficiently comprehensive list of categories to result in a low proportion of deaths classified as “other”	73.6	A global system must include a sufficiently comprehensive list of categories to result in a low proportion of deaths classified as “other”	80	(S9) A global system must include a sufficiently comprehensive list of categories to result in a low proportion of deaths classified as “other”
29. A global system must require the main mechanism of death to be recorded	71.2	A global system must require the main mechanism of death to be recorded	35	-
30. A global system must reduce the percent of death classified as “unknown”	70.6	A global system must reduce the percent of death classified as “unknown”	59	-
31. The causes of death in a global system must map to the ICD	68.6	The causes of death in a global system must map to the ICD	-	-
32. A global system’s causes of death must be mutually exclusive (not overlapping)	66.4	A global system’s causes of death must be mutually exclusive (not overlapping)	-	-
33. A global system must include perinatal deaths for all births after 20 weeks’ gestation	61.5	A global system must include perinatal deaths for all births after 20 weeks’ gestation	-	-
34. A global system must require preventable factors to be recorded	59.6	A global system must require preventable factors to be recorded	-	-
35. A global system must require the degree of certainty for each cause of death to be recorded (unlikely, possibly, probably)	56.9	A global system must require the degree of certainty for each cause of death to be recorded (unlikely, possibly, probably)	-	-
36. A global system must require a principal maternal and a principal fetal/neonatal condition to be classified	55.8	A global system must require a principal maternal and principal fetal/neonatal condition to be classified	-	-
37. A global system must include all perinatal deaths as a result of induced abortions	55.8	A global system must include all perinatal deaths as a result of induced abortions	-	-
38. A global system must be hierarchical	53.7	A global system must be hierarchical	-	-
39. A global system must align with the WHO maternal mortality classification	51.9	A global system must align with the WHO maternal mortality classification	-	-
40. A global system must be clinical rather than pathological	50	A global system must be clinical rather than pathological	-	-
41. A global system should be able to generate classifications from other death classification systems	48.2	A global system should be able to generate classifications from other death classification systems	-	-
42. A global system must not be strictly hierarchical	43.4	A global system must not be strictly hierarchical	-	-
43. A global system must assign causes of death by computer algorithm	39.2	A global system must assign causes of death by computer algorithm	-	-
44. There must be separate global systems for stillbirth and neonatal death	35.9	There must be separate global systems for stillbirth and neonatal death	-	-
45. A global system must use different hierarchy for assigning causes of death for different settings	29.4	A global system must use different hierarchy for assigning causes of death for different settings	-	-
46. A global system must not include associated factors	17	A global system must not include associated factors	-	-

Data are sorted in descending order or agreement in Round 1; System characteristics from Round 3 were divided into Structural (S) and Functional (F) characteristics

*WHO* World Health Organisation, *ICD* International Classification of Disease

### Consultation Round 2: Agreement with proposed system characteristics

The proportion of panel members that agreed or strongly agreed with each proposed system characteristic ranged from 17–98 % (Table 2). Among the system characteristics with the highest agreement were *“A global system must have clear guidelines for use”* (98 %); *“A global system must provide clear definitions for all terms used”* (96 %); *“A global system must be available in multiple languages”* (96 %); and *“A global system must produce data that can be used to inform strategies to prevent perinatal deaths”* (96 %). Among the lower scoring system characteristics were *“A global system must not include associated factors”* (17 %); “*A global system must use different hierarchy for assigning causes of death for different settings”* (29 %); and *“A global system must assign causes of death by computer algorithm”* (39 %). Some of the characteristics were, based on panel feedback, slightly re-worded (see Table 2).

### Consultation Round 3: Retention or discarding of system characteristics

Table 2 presents the proportion of panel members that agreed that each system characteristic from groups 1 and 2 should be retained. Agreement to retain these system characteristics ranged from 35–100 %, with most system characteristics scoring a 70 % or higher agreement-to-retain rate. Based on responses, we defined consensus as an agreement-to-retain rate of 80 % or higher. System characteristics with an agreement-to-retain rate of 80 % or higher were therefore retained, yielding 17 system characteristics. These characteristics were categorised as *Structural*: those that reflected the intent to improve the structure and capacity of the system to achieve its desired objectives (9 characteristics); and *Functional*: those that intended to improve dissemination, function and useability of the system (8 characteristics) (Table 2, column “Round 3 preliminary proposed characteristics”).

We then addressed panel members’ comments to further refine the retained system characteristics. These included characteristic #2 from Round 2 being re-named as the “Overall purpose of the system”. Characteristics were slightly re-worded, merged with a similar characteristic or split into two individual characteristics, to ultimately yield 10 essential structural system characteristics and 7 essential functional characteristics listed below. The overall purpose of system was deemed to be "to produce data that can be used to inform strategies to prevent perinatal deaths”.

#### Structural characteristics (10)

- Accommodates both stillbirths and neonatal deaths.
- Distinguishes antepartum from intrapartum conditions.
- Requires neonatal deaths to be clearly distinguished from stillbirths.
- Requires the single most important factor leading to the death to be recorded.
- Allows associated factors to be recorded and clearly distinguished from causes of death.
- Has a small number of main categories.
- Is multilayered, to accommodate varying levels of available information, in particular the low levels of data available in many LMIC settings.
- Includes a sufficiently comprehensive list of categories to minimise the proportion of deaths classified as "other."
- Ensures cause of death categories are relevant in all settings.
- Includes the level of data available to assign the cause of death (e.g. verbal autopsy only, placental histology, autopsy, etc.).

#### Functional characteristics (7)

- Shows high inter- and intra-rater reliability.
- Has clear guidelines for use and definitions for all terms used.
- Is easy to use.
- Produces data that are easily understood and valued by users.
- Allows easy access to the data by the end-users.
- Is available in different formats including inexpensive ehealth and mhealth options, and in multiple languages.
- Uses rules to ensure valid assignment of cause of death categories.

## Discussion

The current study used a Delphi consensus approach to determine the essential characteristics for a global classification system for perinatal deaths. Central to such a system was “to produce data that can be used to inform strategies to prevent perinatal deaths”, consistent with the goals of the earliest classification systems [17, 18]. We engaged experts in classifying perinatal deaths to specifically identify the characteristics of value to system users to inform the WHO, and collaborating partners, in developing the WHO Application of ICD-10 to perinatal deaths: ICD-Perinatal Mortality (ICD-PM) [13]. Seventeen specific system characteristics were established; 10 relating to structural system properties and 7 relating to functional properties.

The agreed system characteristics were largely consistent with those of de Galan-Roosen and colleagues, who proposed 7 criteria necessary for a perinatal death classification system; *Easy to use by clinicians; Easy to expand in terms of sub-classification; Good level of agreement (low inter-observer variability); Based on clinical factors and necropsy findings including histology of the placenta; Explaining the underlying cause of death; Suitable in stillbirth and neonatal death; and Resulting in high percentage classifiable cases and low percentage of unexplained cases despite a thorough investigation* [12]. Our findings were partially consistent with those of Frøen and colleagues [10], who listed *Compatibility with ICD; Expandability of classifications; Capture of intrapartum events; Ability to differentiate unknown and unexplained events* as desirable system characteristics; and *Capture of placental conditions* as desirable system characteristics*.*

The final system characteristics in the current study did not specifically include the need for a placental category, which has been proposed as essential for any modern perinatal death classification system [6, 10, 17]. Indeed, a lower proportion of unexplained deaths is seen when using systems explicitly recognising placental pathology as a cause of death [6, 19], such as Tulip [20], Codac [21], and the Stockholm system [22]. That including a placental category was not specifically raised by panel members may reflect the argument that such a category is not appropriate for poorly-resourced settings lacking histopathology services. This may also reflect the uncertainty around the links between stillbirth and many placental conditions [23]. However, having a system that can accommodate different levels of data available across differently-resourced settings can address this need. It is important to note that despite the lack of histopathology services in some areas, a placental category (based on clinical findings) may still be relevant, as shown by data from the South African Saving babies 2008–2009 report. Using a clinically based diagnosis (including pre-eclampsia/eclampsia and placental abruption), placenta/placental bed diseases accounted for almost one quarter of perinatal deaths [24].

Shortcomings of the ICD-10, in relation to stillbirths in particular, may explain why compatibility with ICD was less valued by panel members. The ongoing work on WHO Application of ICD-10 to perinatal deaths: ICD-Perinatal Mortality (ICD-PM) aims to “facilitate the consistent collection, analysis and interpretation of information” on perinatal deaths [11], and this process may increase its utility for the understanding and prevention of perinatal deaths [18]. Moreover, the 11th revision of the ICD is currently underway [25], and so formulating ICD-PM provides a unique opportunity to advocate for changes in the existing ICD codes such that the capture of causes of perinatal death are optimised. Work towards refining the conditions and categories is being undertaken [26]. Therefore, although it was not ultimately prioritised by the panel, compatibility with the ICD will be essential for effective global reporting.

The ability to differentiate unknown and unexplained events was also not specifically raised by panel members. Frøen and colleagues’ definition of unexplained stillbirth refers to a death unexpected by history, wherein a cause of death cannot be determined despite thorough autopsy of the infant, and histologic examination of the umbilical cord, placenta, and membranes [27]. The Tulip system makes a distinction between unexplained deaths despite thorough investigation and unexplained deaths with important information missing [20]. Although similar practices have not been widely adopted [6, 10], they unambiguously discriminate truly unexplained deaths from deaths unclassifiable due to under-investigation or lack of information. Therefore, a global perinatal death classification system should at the very least include a clear definition of unexplained stillbirth, and rules to ensure valid use of this category, especially for low- and middle-income countries (LMIC) where under-investigation is more common due to resource shortages. Requiring that the degree of certainty for each cause of death be recorded may also be beneficial in these situations, although this was not ultimately prioritised by the panel.

A global system that includes but clearly distinguishes stillbirths from neonatal deaths was deemed important. This characteristic is essential in order to assess definitional variation, as well as the reporting and registration of perinatal deaths [6]. Requiring that the single most important factor leading to the death be recorded was also highly desirable. This requirement is particularly valuable as it will prevent recording of alleged “causes” that are not underlying causes but rather descriptions of the ultimate mechanism of the death (e.g. “hypoxia”, “asphyxia”), which hold no meaningful information to guide efforts in prevention [10, 17]. Recording associated factors that were clearly distinguished from causes of death was also desirable; a practical characteristic that acknowledges the complexities around the causes of many perinatal deaths, while clearly identifying the key areas for prevention.

There was no consensus on whether the global system should be hierarchical. In Round 2, 54 % of the panel agreed that the system should be hierarchical while 43 % agreed that it should not be strictly hierarchical. The value of hierarchy may lie in greater ease of use and consistency in classification, particularly where multiple factors are present [28] and where expert panel review may not be feasible (e.g. in regions with limited resources). However, criticisms of some hierarchical systems include the potential for inaccurate or misleading causes of death when less important factors are classified solely because they are presented closer to the top of the hierarchy [6, 21]. While a hierarchical system was not explicitly highly valued in this current study, at least some of the purported properties of such an approach were considered key for a system’s global applicability; e.g., that it must include rules to guide users in assigning a cause of death when competing conditions are present.

Another contentious characteristic was the requirement that a principal maternal and a principal fetal/neonatal condition be classified, as recommended in the ICD-10 [29]. Only 56 % of panel members agreed with this requirement in Round 2, but some argued strongly in the final round for retaining the characteristic. Classifying both a maternal and fetal/neonatal condition was seen as essential to retaining all important information to develop effective integrated programs to improve maternal and newborn health. The inherent advantage of assigning maternal and fetal/neonatal conditions to each case is that important information is retained. This could include, for example, indirect or pre-existing maternal conditions such as malaria, sickle cell disease, or congenitally acquired infections. Take the example of antepartum haemorrhage (APH) resulting in preterm birth where the newborn subsequently dies from intraventricular haemorrhage (IVH); both conditions (APH and IVH) are important to prevention through improvements in maternity and newborn care respectively. The incorporation of both a principal maternal and a principal fetal/neonatal condition would correspond broadly to the ‘O’ (obstetric) and ‘P’ (perinatal) groupings of the ICD codes and dovetail with the WHO maternal death classification. However, others felt that this characteristic would introduce difficulties, and/or would not be applicable or relevant in the majority of cases (e.g. where the fetal/neonatal condition is “asphyxia”). Assigning associated conditions (including maternal factors contributing to neonatal deaths) may address this concern.

This is the first study to determine consensus among experts regarding the characteristics important for a globally-acceptable perinatal death classification system. This is a critical formative step towards the development and implementation of such a system, which has been further necessitated by the continued growth of disparate classification systems that produce inconsistent, incongruent data, and hamper understanding of the true causes of perinatal deaths across the world.

The study is strengthened by the application of the Delphi methodology, which lent itself well to first ascertaining essential criteria of a quality global system without the loaded nature of using a pre-prepared list of criteria. Application of the Delphi methodology enabled panel members to respond in each round without coercion from others, and enabled them time to carefully consider their responses in a fashion that is not realistic in traditional group decision-making situations [30]. The Delphi methodology also accommodated for recruitment of panel members covering a wide geographical base and with multi-disciplinary expertise – critical to the topic studied.

The study is limited by the low response rate, which declined with each subsequent round; a finding common to the Delphi methodology [14]. While we were still able to capture a representative sample of global experts in perinatal death classification in Round 3, it is unknown whether the inclusion of more panel members would have meaningfully altered the final results.

## Conclusion

We reached consensus on 17 essential characteristics for a global classification system for the causes of perinatal deaths. To meet the needs of its end-users, the agreed upon list of functional and structural characteristics presented should be taken into consideration when designing and developing such a system.

## Abbreviations

APH, antepartum haemorrhage; ICD, international classification of disease; ICD-PM, international classification of diseases for perinatal mortality; IVH, intraventricular haemorrhage; LMIC, low- and middle-income countries; SDGs, Sustainable Development Goals; WHO, World Health Organisation

## Appendix 1. Round 1 open-ended question

“Please list the criteria you believe must be met by a global system for classification of causes of stillbirth and neonatal death to inform strategies to reduce these deaths. Keep in mind that the system must be usable and useful in low, middle and high-income country settings. You may list as many criteria as you like, but keep to those that you find to be most important. Please be as specific as possible.”
